# Nuclear receptor corepressor (NCoR) is a positive prognosticator for cervical cancer

**DOI:** 10.1007/s00404-021-06053-3

**Published:** 2021-04-16

**Authors:** Daniel Beilner, Christina Kuhn, Bernd P. Kost, Theresa Vilsmaier, Aurelia Vattai, Till Kaltofen, Sven Mahner, Elisa Schmoeckel, Christian Dannecker, Julia Jückstock, Doris Mayr, Udo Jeschke, Helene Hildegard Heidegger

**Affiliations:** 1 Department of Obstetrics and Gynaecology, Ludwig-Maximilians-University of Munich, Maistrasse 11, 80337 Munich, Germany; 2 Department of Pathology, LMU Munich, Thalkirchner Street 56, 80337 Munich, Germany; 3 Department of Obstetrics and Gynaecology, University Hospital Augsburg, Stenglinstr. 2, 86156 Augsburg, Germany

**Keywords:** NCoR, Epigenetic, Cervical cancer, Prognosis, Immunohistochemistry

## Abstract

**Purpose:**

Enzymes with epigenetic functions play an essential part in development of cancer. However, the significance of epigenetic changes in cervical carcinoma as a prognostic factor has not been fully investigated. Nuclear receptor corepressor (NCoR) presents itself as a potentially important element for epigenetic modification and as a potential prognostic aspect in cervical cancer.

**Methods:**

By immunohistochemical staining of 250 tumor samples, the expression strength of NCoR was measured and evaluated by immunoreactive score (IRS) in the nucleus and cytoplasm.

**Results:**

A low expression of NCoR in our patients was a disadvantage in overall survival. Expression of NCoR was negatively correlated with viral oncoprotein E6, acetylated histone H3 acetyl K9 and FIGO status, and positively correlated to p53.

**Conclusions:**

Our study has identified epigenetic modification of tumor cells thus seems to be of relevance in cervical cancer as well for diagnosis, as a marker or as a potential therapeutic target in patients with advanced cervical carcinoma.

## Introduction

Cervical cancer is the fourth most frequent cancer in women worldwide with about 570,000 new cases in 2018, and this represents 7.5% of all female cancer deaths. In less developed regions, cervical cancer is the second most common cancer in women living in this regions, and is about 84% of the new cases worldwide [1]. In the developed world screening for cervical cancer including cervical cytology, human papillomavirus (HPV) or both as well as HPV vaccination has strongly reduced the incidence of cervical cancer [2]. The two main types of cervical cancer are squamous cell cervical cancer comprising 80–85% and adenocarcinoma comprising 15–20% [3, 4].

The persistent infection with high-risk human papillomavirus (HR-HPV) is the leading cause of cervical cancer. Papillomaviruses are double-stranded, circular DNA viruses. More than 150 HPV types are identified, and only some of them can infect the cervix, named HR-HPV [5]. The group of low-risk HPV types including HPV 6 and HPV 11 is associated with benign anogenital warts that infrequently progress to cancer and the group of high-risk HPV types including HPV 16 and HPV 18 is associated with lesions that are high risk for malignant progression and for cervical cancer [6].

The two oncoproteins E6 and E7, which are both involved in the cellular transformation, are encoded by the high-risk HPV types [7]. E6 and E7 interact with the regulatory proteins in cells like p53 and the retinoblastoma gene (Rb). The E6 protein of HPV 16 is able to bind the cellular p53 and the E7 protein is able to bind the retinoblastoma tumor suppressor gene product, so they modulate the tumor suppressors and contribute also to carcinogenesis [6, 8]. Further studies are needed to understand the complex mechanism that are modulated by HPV E6 and E7.

For the gene expression, many transcription factors and cofactors are needed. The cofactors can activate (the coactivators) or repress (the corepressors) gene transcription. One of the first identified are the nuclear receptor corepressor (CoRs), which include NCoR (nuclear receptor corepressor). Deregulated function of NCoR has been found in many types of cancers [9]. NCoR levels, for example, are downregulated in invasive ductal breast carcinomas [10]. The important role of NCoR in cancer development such as prostate cancer [11] or leukaemia [12] has been suggested in recent studies. In addition, we could recently show that the NCoR-related proteins RIP140 and LCoR are independent markers for poor prognosis in cervical cancer [13].

To find out the role of NCoR in cervical cancer, more studies are needed, so the aim of our study was to revise the significance of NCoR as a prognostic factor in cervical cancer.

## Materials and methods

### Patients

Our analysis included 250 paraffin-embedded cervical cancer samples. The 250 patients had undergone surgery at the Department of Obstetrics and Gynaecology of the Ludwig-Maximilians University of Munich (LMU) between 1993 and 2002 to include only patients without HPV vaccination. The median age of the group was 48.0 with a range from 22 to 83 years. The study included only the two most frequent histological subtypes, squamous cell carcinoma and adenocarcinoma, due to the low number of other cases. For the positive control of the staining, we used a placenta tissue supplied from the Department of Obstetrics and Gynaecology of the LMU. Clinical data for statistical analyses and the follow-up data were provided by the Munich Cancer Registry and recruited from medical records (Table 1).

**Table 1:** Clinical and pathological parameters of patients included in this study

Clinical parameter	No./total no.	%
Age		
≤ 50 years	141/250	56.4
> 50 years	105/250	42.0
N/A	4/250	1.6
pN		
Negative	151/250	60.4
Positive	97/250	38.8
N/A	2/250	0.8
pT		
T1	111/250	44.4
T2	128/250	51.2
T3/4	9/250	3.6
FIGO		
I	64/250	25.6
II	48/250	19.2
III	37/250	14.8
IV	7/250	2.8
N/A	94/250	37.6
Grading		
G1	20/250	8.0
G2	143/250	57.2
G3	78/250	32.2
N/A	9/250	3.6
Histological subtype		
Squamous carcinoma	202/250	80.8
Adenocarcinoma	48/250	19.2
Recurrence (within 235 months)		
None	190/250	76.0
≥ 1	58/250	23.2
N/A	2/250	0.8

### Immunohistochemistry

Samples of 250 patients were formalin-fixed and paraffin-embedded. 3 µm tissue slices were obtained from the paraffin material and prepared on microscope slides. In the first step, the slides were pre-treated with Roticlear for deparaffinization followed by washing in 100% Ethanol. After blocking the endogenous peroxidase with 3% methanol/H_2_O_2_, the samples were treated in a descending alcohol series for rehydration and washed in distilled water. In a pressure cooker, the samples were heat-treated in a sodium-citrate buffer (pH = 6.0) for 5 min by up to 100 °C. Then, the samples were first cleaned in distilled water followed by a washing step in PBS-buffer. Before incubating the samples with the primary antibody Anti-NCoR (rabbit IgG, company: abcam, order number: ab3482) for 16 h at 4 °C, all slides were treated with a blocking solution to avoid unspecific hydrophobic binding. After the incubation, the slides were washed in PBS-buffer and covered with a post-block solution. After one more washing in PBS-buffer and applying the HRP-polymer (mouse/rabbit, company: Zytomed, order number: POLHRP-100), the substrate staining with DAB (company: Dako, order number: K3468) was performed. Immediately, following the counterstaining by Hemalum, in the final step, the tissue was dehydrated in a rising alcohol series and finally covered. Placenta tissue was used as a positive control. After the staining, the slides were evaluated by the immunoreactive score (IRS) with an optical microscope. For microscope images, a light microscope “Immunohistochemistry Type 307-148.001 512 686” (company: Leitz, Germany) and “IH-Camera 3CCD Colour Video Camera” (company: Fissler) was used. For image acquisition, “Discuss” software (Version 4) was used. Time and space resolution data are 760 × 574 pixels, and bit depth is 24 mm. According to the expression, the nucleus and cytoplasm of the cervical cancer cells were rated from 0 (no expression) to 12 (very high expression). IRS was calculated from the intensity of the staining (0 = not stained; 1 = low intensity; 2 = moderate intensity; 3 = high intensity) multiplied by the percentage of stained cells (0 = not stained; 1 = 1–10%; 2 = 11–50%; 3 = 51–80%; 4 ≥ 80%).

### Statistics

For statistical analysis, we created a database using IBM SPSS Statistics version 25 (Amrok, NY, USA). The cumulative survival time was calculated and visualized in Kaplan–Meier curves to compare survival rates. For all statistical results, *p* was required to be < 0.05.

## Results

### NCoR staining in cervical cancer

**Fig. 1:**
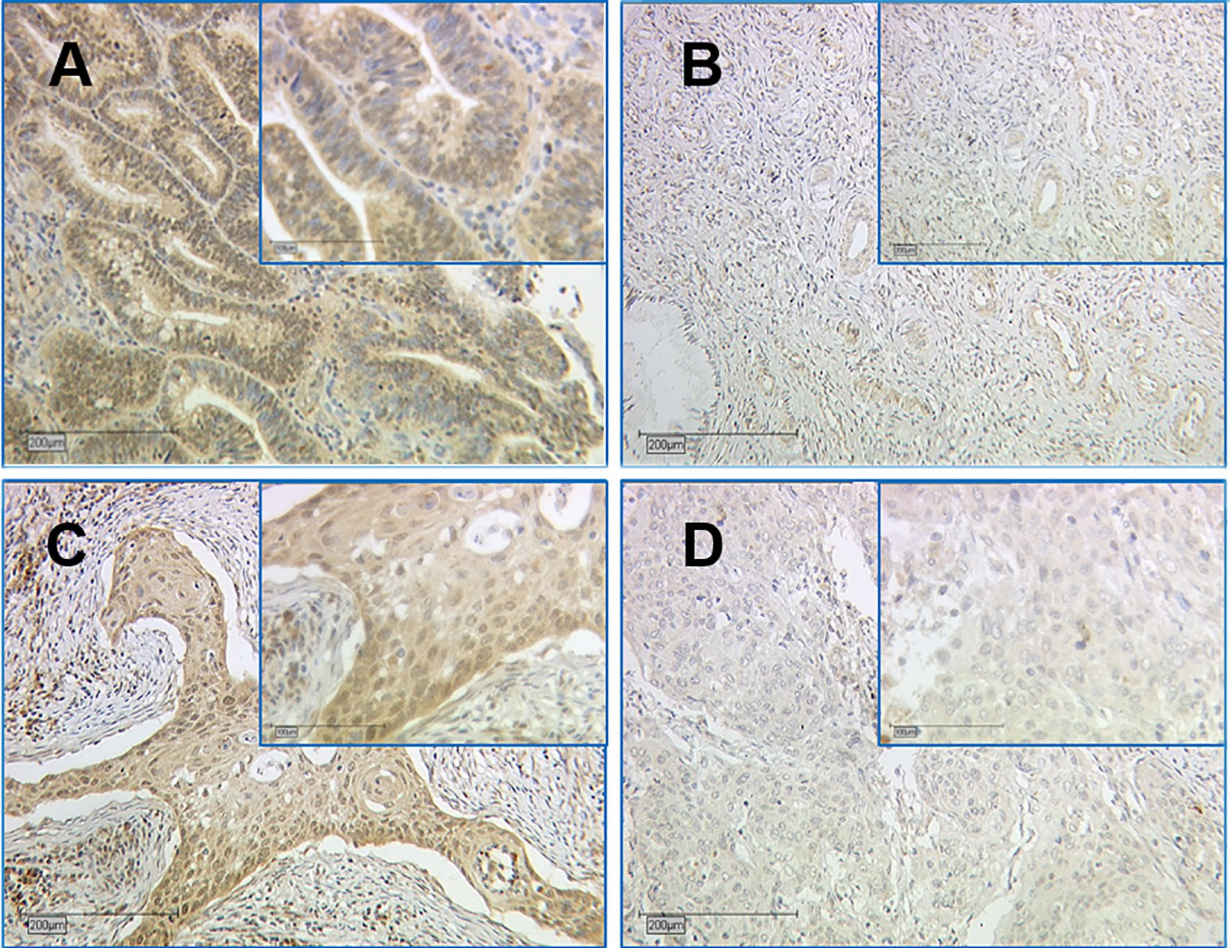
NCoR staining in cervical cancer. Cytoplasm was scored for image 1a with IRS of 12 (high intensity, ≥ 80% stained cells), image 1b with IRS of 2 (low intensity, 11–50% stained cells), image 1c with IRS of 12 (high intensity, ≥ 80% stained cells) and image 1d with IRS of 4 (low intensity, ≥ 80% stained cells). Nucleus was rated for image **a** with IRS of 12 (high intensity, ≥ 80% stained cells), image **b** with IRS of 1 (low intensity, 1–10% stained cells), image **c** with IRS of 12 (high intensity, ≥ 80% stained cells) and image 1d with IRS of 3 (low intensity, 51–80% stained cells). Image **a**/**b** were histologically diagnosed as adenocarcinoma compared to image **c**/**d** as squamosa cell carcinoma. 10 × magnification was used for overview images (200 µm scale bar) with additional 25 × magnification (100 µm scale bar)

**Fig. 2:**
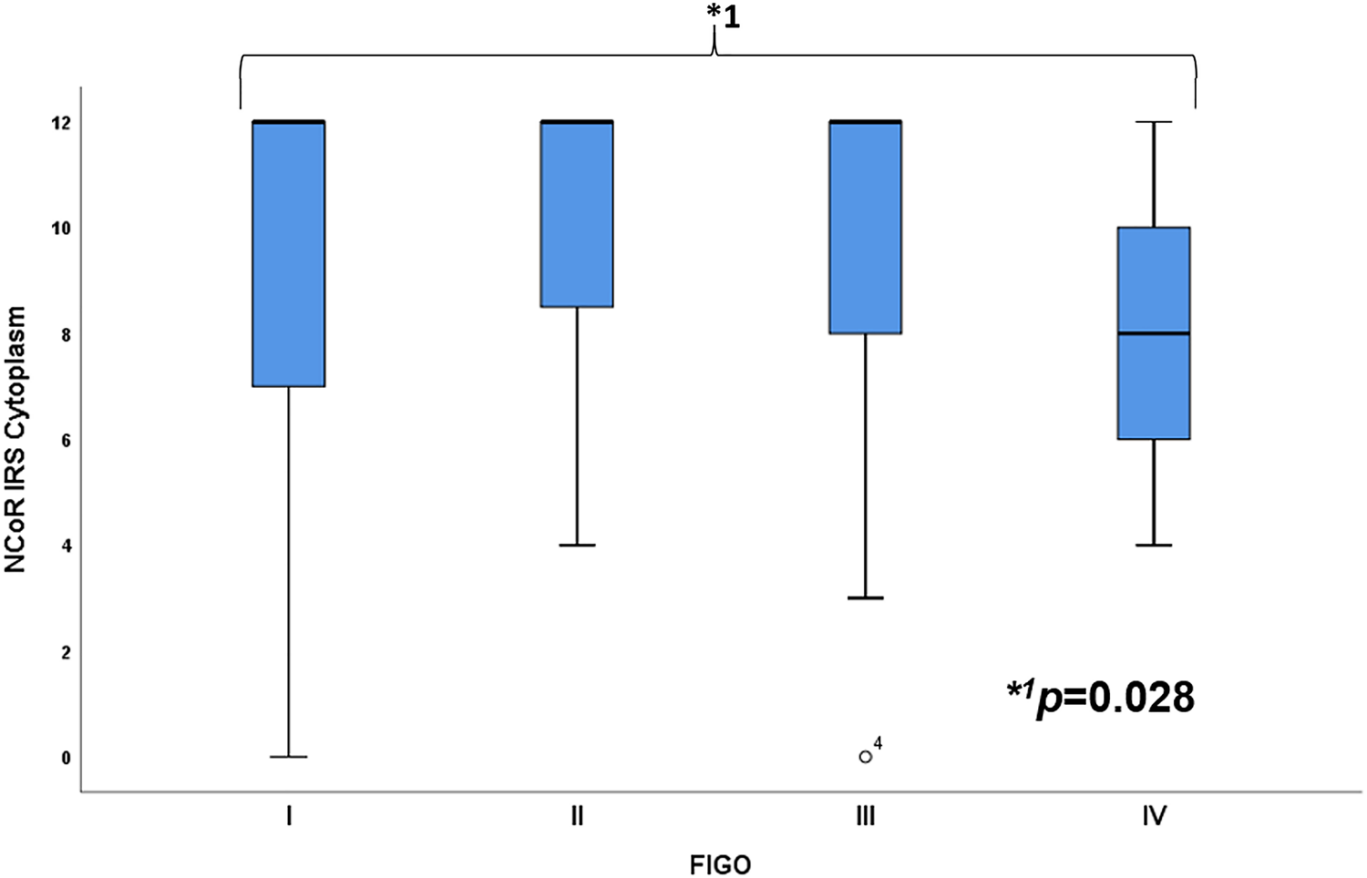
Boxplot summary for FIGO. **a** Boxplots for cytoplasmic IRS of NCoR and FIGO with a median IRS of 12 for FIGO I–III and a median IRS of 8 for FIGO IV. The asterisk indicates significant lower expression of NCoR in patients with advanced FIGO classification

The median cytoplasmic IRS of the staining was 12 compared to a nuclear median IRS of 8. 1.7% of the patients had no expression in the cytoplasm and 0.4% no detectable staining in the nucleus. 10.3% showed a low expression (IRS = 1–5), while 89.7% presented a high expression (IRS 6–12) in the cytoplasm. In comparison with the nucleus with 17.8% with a low and 82.2% with a high expression, the median nuclear IRS for squamous carcinoma was 8 and adenocarcinoma was 8.5. For both histological subtypes, the median cytoplasmic IRS was 12 (Fig. 1). The median IRS in the cytoplasm for grading from G1 to G3 was 12, and the median nuclear IRS was 8 for each grading. Patients with lymph node metastasis (N +) and without lymph-node metastasis (N−) had a median IRS of 12 in the cytoplasm and a nuclear IRS of 8 for N + and N−. For FIGO I–III, the median IRS was 12, while FIGO IV showed a median IRS of 8 in the cytoplasm (Fig. 2). In comparison, the nucleus was stained with a median IRS of 8 for FIGO I–III and the median IRS of 3 for FIGO IV. Tumour size showed in the cytoplasm a median IRS of 12 for T1 and T2, while the median IRS of T3/4 was 8. The nucleus showed a median IRS of 8 for T-status T1 and T2 compared to a median IRS of 6 for tumor size T3/4. Table 2 shows staining results for NCoR in summary.

**Table 2:** Median IRS of NCoR for low and high expression, histological subtype, grading, T-status, N-status and FIGO classification

NCoR	Cytoplasm	Nucleus
Median IRS	12	8
Expression		
No expression	1.7%	0.4%
IRS 1–5	10.3%	17.8%
IRS 6–12	89.7%	82.2%
Histological subtype (IRS)		
Squamous carcinoma	12	8
Adenocarcinoma	12	8.5
Grading		
G1	12	8
G2	12	8
G3	12	8
T-status		
T1	12	8
T2	12	8
T3/4	8	6
N-status		
N(+)	12	8
N(−)	12	8
FIGO		
I	12	8
II	12	8
III	12	8
IV	8	3

### Correlation analyses of NCoR staining with other parameters in cervical cancer

We found statistically significant negative correlations of NCoR with E6 (*p* = 0.003), NCoR with FIGO status (*p* = 0.004), and NCoR with acetylated histone H3 acetyl K9 (*p* = 0.003). A positive correlation was found between NCoR and tumor suppressor p53 (*p* = 0.01). The results of the correlational analysis are presented in Table 3.

**Table 3:** Correlation analyses of NCoR staining with other parameters in cervical cancer

	E6	FIGO	H3 acetyl K9	p53
Correlation	− 0.193	− 0.184	− 0.193	0.166
*p*	0.003	0.004	0.003	0.01

There are statistically significant negative correlations of NCoR with E6 (*p* = 0.003), with FIGO status (*p* = 0.004) and with acetylated histone H3 acetyl K9 (*p* = 0.003). A positive correlation was found between NCoR and tumour suppressor p53 (*p* = 0.01)

### NCoR expression in cervical cancer regarding survival

**Fig. 3:**
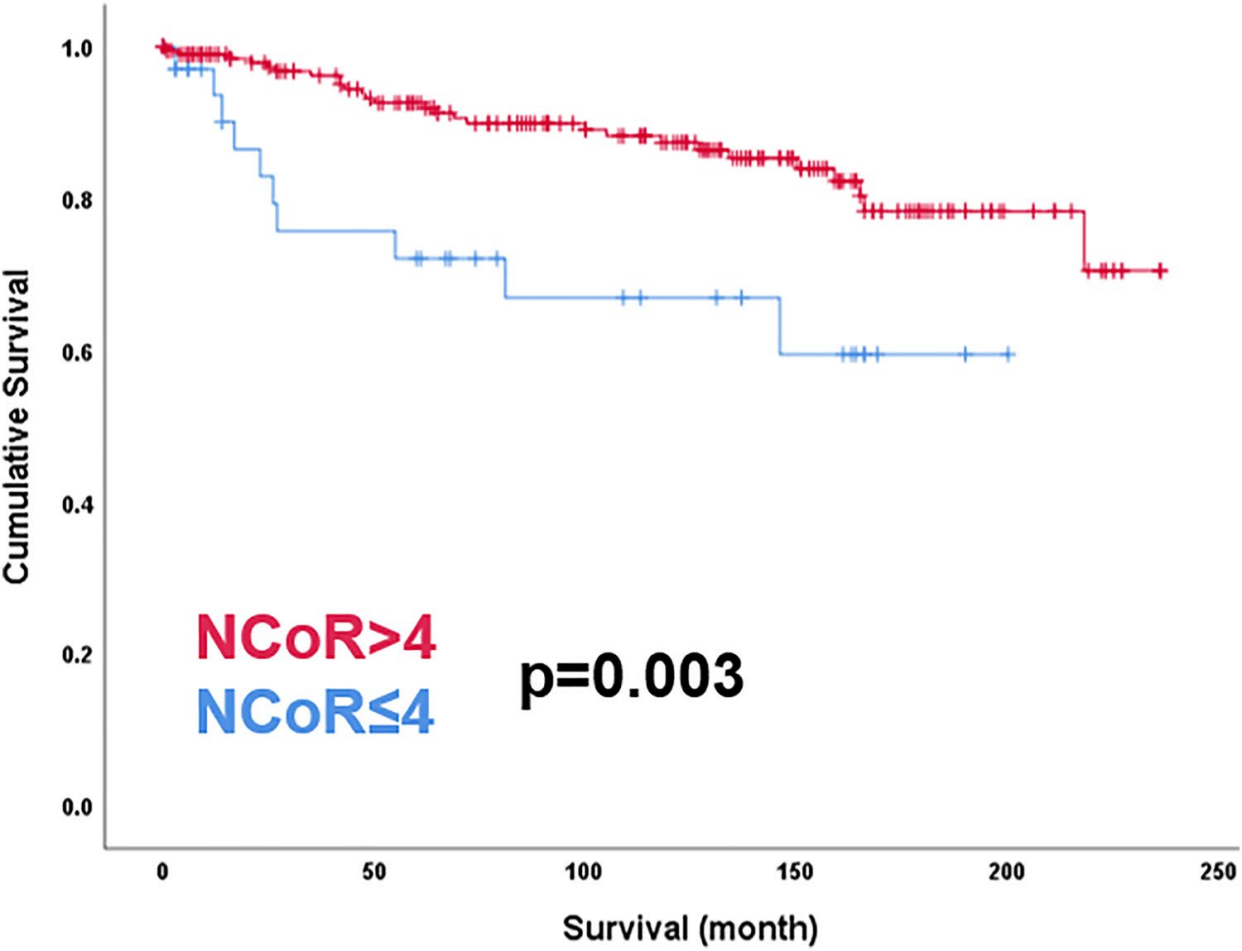
Kaplan–Meier analyses for relapse-free survival. High nuclear NCoR expression (IRS > 4; red) compared to low nuclear expression (IRS ≤ 4; blue) regarding relapse-free survival (*p* = 0.003)

Finally, further investigations of prognosis revealed a significant disadvantage of patients with a low nuclear expression of NCoR (IRS < 4) in overall survival (*p* = 0.003). As presented in the Kaplan–Meier curve, patients in our study group with a low expression of NCoR were correlated with poor prognosis in overall survival (Fig. 3).

### Cox regression

For further analysis of our collected data, we performed a multivariate cox-regression to detect independent histological parameters for survival in our study group. For overall survival, the histological subtype (*p* = 0.007), pT-status (*p* = 0.016), and nuclear NCoR expression were independent prognosticators (Table 4).

**Table 4:** Cox regression of clinic pathological variables regarding progress-free survival, histological subtype (*p* = 0.007), pT-status (*p* = 0.016) and nuclear NCoR expression were independent prognosticators

Variable	Significance	Hazard ratio of Exp (B)	Lower 95% CI of Exp (B)	Upper 95% CI of Exp (B)
Histology	0.007	3.618	1.413	9.266
pT	0.016	3.311	1.252	8.753
pN	0.858	0.990	0.883	1.109
FIGO	0.718	0.880	0.439	1.763
Grading	0.369	1.435	0.652	3.155
NCoR nucleus (IRS > 4)	0.010	0.279	0.106	0.735

## Discussion

Our results provide further evidence that epigenetic modulations might play a role in cervical cancer. In this study, we observed that a lower nuclear expression of NCoR was related to a significant disadvantage in overall survival. In further analysis of this patient collective, we identified a negative correlation of NCoR to HPV E6 oncoprotein [14, 15]. Our results suggest that connections of NCoR and E6 oncoprotein for patients diagnosed with cervical cancer which might be of relevance for better survival.

Disruption of epigenomic control can be classified as an enabling characteristic of cancer cells and essential for mutation and malignancy [16–18]. Epigenetic modifications have a strong influence on the expression of the DNA by regulating the biochemical and structural properties of chromatin [19]. One described mechanism in the complex field of gen regulation is the acetylation of histones by enzymes [20–22]. Increased acetylation of histone lysine residues is associated with increased chromatin accessibility and gene expression [23]. This process of histone acetylation and deacetylation has a major role in modulating chromatin accessibility during transcription, replication, and repair [17, 24, 25]. NCoR is a well-studied regulator of gene expression that assembles a multi-protein complex and binds histone deacetylase HDAC3 [26]. Our results support the idea of the NCoR/HCD3 complex with crucial deacetylase functions presented through a negative correlation of NCoR with Histone H3 acetyl K9 from the previous investigations on this specimen [27]. This study is supporting the idea from the previous observations of NCoR and associated histone deacetylases to be recruited to target genes by interaction with nuclear receptors and other transcription factors, causing chromatin compaction and blocking transcription [28–31]. It is proposed that disruption of cell cycle functions of NCoR has dramatic consequences for the regulation of chromatin structure and genomic stability [26]. Our study provides additional support for crucial cell cycle functions of NCoR also in cervical cancer cells.

Further analysis of our patient’s collective presented a negative correlation between the expression of NCoR and HPV E6 oncoprotein [14, 15]. This suggests that a link may exist between both proteins. Prior studies have noted the importance of E6 and E7 in proliferating cells as a trigger factor for HPV-induced malignant transformation [32]. It was shown that the expression of HPV-16 E6 disturbs the genomic structure and induces numerical and structural chromosome instability [33] through proteolytic degradation of p53 [34] or direct DNA modification such as viral DNA integration or methylation of viral promoter regions [35]. It is not surprising that high-risk HPV E6 has been reported to interact with a variety of epigenetic enzymes including DNA methylases and histone-modifying enzymes [36]. Our findings are in accord with recent studies indicating that E6 oncoprotein leads to proteolytic degradation of p53 provided by a negative correlation of NCoR with E6 and a positive correlation of NCoR with p53.

The negative correlation of NCoR and HPV E6 oncoprotein further supports the idea of E6 and E7 to inhibit the binding of the histone-deacetylase-3/NCoR complex to the COX-2 promoter [37]. Several reports have shown that prostaglandin derived from COX-2 can stimulate cell proliferation and angiogenesis while inhibiting apoptosis [38–41]. These results provide further support for the hypothesis that HPV oncoproteins can modulate the function of NCoR.

These findings raise intriguing questions regarding the function of NCoR as a gene silencer to replication and transcription in cervical cancer as a defense mechanism. A further study with more focus on the functional significance of NCoR in cervical cancer cells is, therefore, suggested. This is an important issue for future research to develop a full picture of NCoR as a highly interesting target in diagnostic and pharmaceutical treatment.

The present research aimed to examine the significance of NCoR as a prognostic factor in cervical cancer. The results present a low expression of NCoR with a significant disadvantage in overall survival. Taken together, this study strengthens the idea that the role of epigenetic modifications through enzymes plays also a crucial role in cervical cancer and provides a deeper insight into the influence of HPV E6 oncoprotein in cervical cancer. A greater focus on NCoR could produce interesting findings for better diagnostics and therapy in cervical cancer patients.

## Declarations

### Availability of data and materials

The data sets used during the current study are available from the corresponding author on reasonable request.
